# Gut microbiota variation in captive tortoises is associated with host species, trophic category, and growth rate

**DOI:** 10.3389/fmicb.2026.1861012

**Published:** 2026-09-10

**Authors:** Kimberly Vanous, Connie A. Rojas, Carly Pomeroy, Holly H. Ganz

**Affiliations:** 1 Big and Small Tortoise Rescue and Sanctuary, Ames, IA, United States; 2 Animal Biome, Oakland, CA, United States

**Keywords:** 16S rRNA, age, growth rate, gut bacterial community, red-footed tortoise, Russian tortoise, microbiome, Testudinidae

## Abstract

Despite the ecological and conservation importance of tortoises, their gut bacterial microbiotas remain poorly characterized, particularly across species in captivity. Associations between gut microbiota composition and tortoise age, sex, and growth rate are also poorly understood. Here, we use full-length 16S rRNA gene sequencing to characterize the gut microbiotas of 55 captive tortoises, representing seven species: red-footed tortoise (*Chelonoidis carbonarius*), yellow-footed tortoise (*Chelonoidis denticulatus*), Mojave desert tortoise (*Gopherus agassizii*), Texas tortoise (*Gopherus berlandieri*), leopard tortoise (*Stigmochelys pardalis*), African spurred tortoise (*Centrochelys sulcata*), and Russian tortoise (*Testudo horsfieldii*). By examining individuals maintained under consistent environmental conditions, we reduced large-scale environmental variation and evaluated associations with host characteristics. Across species, we identified a core set of 18 bacterial taxa, including cellulose degraders (*Cellulosilyticum ruminicola* and *Ruminococcus champanellensis*) and fatty-acid producers (*Clostridium butyricum*, and members of the families *Lachnospiraceae* and *Christensenellaceae*). Despite this shared bacterial core, microbiota composition differed by host species, age, and trophic category (herbivore vs. omnivore). Additionally, the relative abundances of two taxa—*R. champanellensis*, an important fiber degrader, and *Anaerocella delicata*, a propionate and acetate producer—were associated with variation in tortoise growth rate. Collectively, our findings suggest that although captive tortoises share a common microbial core, host species and digestive physiology shape microbiota structure, while specific bacterial taxa are associated with variation in growth rate.

## Introduction

Gut bacterial microbiotas, hereafter referred to as the “gut microbiota,” are defined here as the bacteria inhabiting the gastrointestinal tract. These communities are shaped by complex interactions between environmental and host-associated factors, making it challenging to disentangle their relative contributions. In many studies, particularly those comparing individuals across geographic locations, environmental variation has been identified as a dominant driver of microbiota composition (Ley et al., 2008; Colston and Jackson, 2016). For example, recent work in Aldabra giant tortoises (*Aldabrachelys gigantea*) (Zakaria et al., 2025), domestic horses (*Equus ferus caballus*) (Ang et al., 2022), and field mice (*Apodemus draco*) (Yun et al., 2026) demonstrated that microbiota composition varies strongly with geographic location. Such large-scale environmental effects can obscure more subtle associations between host traits and microbiota variation.

Examining animals within a shared environment, thereby minimizing environmental variation, provides a complementary approach to disentangling finer-scale microbiota associations. Within such settings, microbiota composition may be structured by host-associated factors including species (Zoelzer et al., 2021; Lan et al., 2022; Hoffbeck et al., 2023), diet (McKenzie et al., 2017; McKenney et al., 2018; Jiang et al., 2017), and age (Chen et al., 2020; López-Espinoza et al., 2025), while additional variation arises from individual-level influences such as early-life history (Beurel and Nemeroff, 2024; Dong and Gupta, 2019), legacy effects of prior environmental exposure (Moustafa et al., 2021; Forehand et al., 2025), stochastic microbial colonization (Keady et al., 2023; Frazier et al., 2025), and behaviors such as coprophagy (Videvall et al., 2023; Colston, 2017) that facilitate microbial transfer between individuals. Sanctuaries, in particular, represent a useful study system because they house multiple species within a single location while also encompassing individuals with diverse life histories and health conditions. As a result, these populations likely share a common environmental microbial backdrop but may showcase both structured and individual-level variation in microbiota composition.

Within this context, captive tortoises provide an opportunity to examine associations between host traits and microbiota variation. Tortoises encompass a diverse group of hindgut fermenting herbivores adapted to a wide range of environments and dietary niches (Vlachos, 2018; Crumly, 1984), resulting in variation in feeding strategies from predominantly herbivorous to more omnivorous diets (Figueras et al., 2021; Richardson and Stiling, 2019). They also vary substantially in life-history traits, including growth rate, body size, and longevity (Stott et al., 2024). Across this diversity, tortoises rely heavily on their gut microbiota to process complex dietary substrates and support host metabolism, particularly through the fermentation of plant material into short-chain fatty acids that contribute to energy balance (Blair et al., 2025; Bouchard and Bjorndal, 2006; Zhou et al., 2025). Thus, variation in gut microbiota composition has the potential to translate into differences in host growth and condition.

Understanding how microbiota variation relates to host growth is particularly relevant in captive tortoises, where growth rates often vary substantially among individuals. This variability is a common and often frustrating challenge for owners and caretakers, as tortoises of the same species maintained under similar conditions can exhibit markedly different growth patterns with no clear explanation. While diet and husbandry are known contributors, substantial unexplained variation frequently remains, suggesting that additional biological factors may be involved.

To address these gaps, we used full-length 16S rRNA gene sequencing to survey the gut bacterial communities of 55 captive tortoises spanning seven species within the order *Testudine*. The species were the red-footed tortoise (*N* = 25), yellow-footed tortoise (*N* = 2), Mojave desert tortoise (*N* = 2), Texas tortoise (*N* = 2), leopard tortoise (*N* = 6), African spurred tortoise (*N* = 4), and Russian tortoise (*N* = 14; Table 1, Supplementary Table S1). We identified the core bacterial taxa shared across individuals, evaluated associations between microbiota variation and host traits (age, sex, growth rate, trophic category, and species), and uncovered significant relationships between fiber-degrading bacteria and host growth rate.

**Table 1:** List of tortoise study species and their associated metadata.

Common name	Latin name	Diet	Age (yrs) (mean; range)	Sex (F, M)	Weight (kg) (mean; range)	Samples (*N*)
Red-footed tortoise	*Chelonoidis carbonarius*	Omnivore	11.7; 3–20	14F, 11M	4.55; 0.9–9.27	25
Yellow-footed tortoise	*Chelonoidis denticulatus*	Omnivore	15; 10–20	1F, 1M	8.32; 6.8–9.8	2
Mojave Desert tortoise	*Gopherus agassizii*	Herbivore	46.5; 30–60	1F, 1M	3.5; 2.4–4.5	2
Texas tortoise	*Gopherus berlandieri*	Herbivore	5; 5–5	0F, 2M	0.75; 0.62–0.88	2
Leopard tortoise	*Stigmochelys pardalis*	Herbivore	6.83; 4–9	4F, 2M	6.9; 1.01–15.5	6
African Spurred tortoise	*Centrochelys sulcata*	Herbivore	12.5; 5–15	1F, 3M	20.05; 5.4–42.2	4
Russian tortoise	*Testudo horsfieldii*	Herbivore	17.64; 7–35	9F, 5M	0.90; 0.42–1.52	14

## Materials and methods

### Site description

This study was conducted at Big and Small Tortoise Rescue and Sanctuary (Ames, IA, United States), a nonprofit facility housing a diverse population of captive tortoises of multiple species. At the time of sampling, approximately 70 tortoises representing 12 species were maintained at the facility. Tortoises were primarily surrendered by private owners and arrived with varying histories and health conditions. Many tortoises are long-term residents, being housed on site for 10+ years.

Tortoises are grouped by species and maintained in separate enclosures to reduce interspecies microbial exchange, although the facility does not operate as a strict quarantine environment. Housing conditions among these groups were based on seasonal requirements. Three enclosures are maintained for each group—outdoor in summer, greenhouse for spring/fall, and indoors for winter. Climate-controlled indoor environments were maintained with ambient temperatures at least above 21 °C. Basking areas with artificial UVB in the species-appropriate Ferguson zones (Baines et al., 2016) were provided during winter months (typically no more than 2 months annually).

Tortoises were sampled during the summer months (June–August 2025) under typical seasonal conditions for central Iowa. During this period, tortoises were maintained outdoors during daytime hours with access to natural environmental conditions, including ambient temperatures generally ranging from approximately 27–29 °C. Overnight, individuals were housed within a greenhouse environment maintained at approximately 21 °C, limiting exposure to lower nocturnal temperatures. Animals were maintained under consistent husbandry practices, including standardized feeding routines and access to water. Base diets for all species consisted primarily of native grasses and weeds, along with purchased greens such as collard, turnip, mustard greens, kale, red and green leaf lettuce, and romaine. Commercial pellets were also fed as supplementation at least once a week (Formula 5 m21, Mazuri Exotic Animal Nutrition, Gray Summit, MO, United States). Further variation in diets reflected natural feeding strategies (e.g., herbivorous vs. more omnivorous tendencies). Specifically, red-footed and yellow-footed tortoises were additionally fed protein once a month via commercial omnivore gel diets (Repashy Superfoods, Oceanside, CA, United States), as well as a variety of fruits (excluding citrus) at least once a week.

### Metadata collection

Individual tortoises were identified based on unique morphological features and/or established naming records maintained by the sanctuary. Metadata collected for each individual included species, age (estimated when necessary), sex, trophic category classification, and growth rate.

When exact ages were not available, estimates were derived from a combination of known history (time at the sanctuary), intake records from previous owners, and assessment of shell wear and condition. Sex was determined based on established secondary sexual characteristics, including tail length, plastron concavity, and anal scute morphology, with size, body shape, and behavioral traits used as supporting indicators when applicable. Trophic classification was assigned based on species-specific feeding ecology and established husbandry practices, with individuals categorized as herbivorous or omnivorous.

Growth rates were calculated from monthly logs spanning January 2019 through July 2025 using longitudinal weight measurements collected for each individual. Raw growth rate was defined as the change in body mass over time, calculated as (current weight—initial weight) divided by the duration between measurements (in years). To enable comparisons across species and developmental stages, growth rate was further normalized by dividing the raw growth rate by both current body weight and age. This normalization accounts for differences in species size discrepancies and the expectation of higher growth rates in younger individuals.

### Sample collection

Fecal samples were collected from 55 tortoises during June, July, and August 2025, directly after defecation, to ensure the host individual was accurately noted. A nail-size amount of fecal matter was taken from the middle of the stool and placed into two 2 mL screw-cap tubes containing Nucleic Acid Preservation (NAP) buffer (Animal Biome, CA, United States) to stabilize the bacterial DNA (Camacho-Sanchez et al., 2013). The samples were shipped to Animal Biome’s laboratory for DNA extraction and sequencing.

### DNA extraction and sequencing

Genomic DNA was isolated from tortoise fecal samples (*N* = 55) using the QIAGEN DNeasy Powersoil Pro Kit using the automated QIAcube HT system (QIAGEN, CA, United States). The full-length 16S rRNA gene for bacteria was amplified with primers 27F (5′-AGRGTTYGATYMTGGCTCAG-3′) and 1492R (5′-RGYTACCTTGTTACGACTT-3′). PCR reactions were prepared using KAPA HiFi HotStart ReadyMix (12.5 μL; KAPA Biosystems, MA, United States) with 3 μL of each primer (2.5 μM) and 2 μL of template DNA in a final volume of 25 μL. Amplification involved an initial denaturation at 95 °C for 3 min, followed by 25 cycles of denaturation at 95 °C for 30 s and annealing at 57 °C for 30 s, with a final extension at 72 °C for 1 min. Purified amplicons were then sequenced on a PacBio Sequel IIe platform (Pacific Biosciences, CA, United States). To monitor sequencing quality and contamination, each run included the ZymoBIOMICS Microbial Community DNA Standard (Zymo Research, Irvine, CA) as a positive control and PCR-grade water as a negative control.

Samples spanned two sequencing runs but tortoise gut bacterial community composition remained consistent across runs, with no evidence of batch effects (PERMANOVA *F* = 1.37, *R*^2^ = 0.024, *p* = 0.151; Supplementary Figure S1).

### Bioinformatics processing of 16S rRNA reads

PacBio reads were trimmed, denoised, and classified within QIIME 2 (Bolyen et al., 2019), as detailed in Rojas et al. (2024). The SILVA (v.138.1 NR99) bacterial reference database (Quast et al., 2013) was used for classification, along with the QIIME sklearn and VSEARCH classifier tools (Bokulich et al., 2018; Rognes et al., 2016). The table of bacterial ASV counts was aggregated at the species level (Supplementary Table S2) and imported into the R statistical software program (v.4.3.0) (R Core Team, 2021) for subsequent statistical analyses and visualizations.

### Gut microbiota statistics

The core microbiota was defined as the bacterial taxa detected in 70% of tortoise samples at a minimum average relative abundance of 0.5% across samples. This cutoff captured taxa corresponding to presence in at least two-thirds of individuals, representing a robust and consistently shared community. A more stringent cutoff (e.g., 90%) would result in only seven taxa and likely exclude ecologically meaningful members. There is no unanimously agreed on prevalence threshold for the core microbiota and in animal studies it can vary from 33% (Rojas et al., 2024) to 90% (Grieneisen et al., 2017).

Composition plots of the most abundant bacterial species (mean relative abundance >1%) were generated using the ggplot2 R package.

Estimates of alpha-diversity, including Observed Richness and Shannon Diversity were calculated using the *phyloseq* (McMurdie and Holmes, 2013) R package, while Pielou’s evenness was estimated with the R *microbiome* package (v1.22.0) (Lahti and Shetty, 2018). Associations between alpha diversity and tortoise age, sex, growth rate (normalized by age and weight), and trophic category were evaluated using generalized linear models (GLMs) implemented in the R *stats* package (v4.5.1) (R Core Team, 2021). A separate model assessed the effect of host species and only included samples from tortoise species with the largest sample sizes: red-footed, Russian, and leopard tortoises. Model assumptions were evaluated by inspecting diagnostic plots (including Q-Q plots and residuals versus fitted values), and Levene’s test for homogeneity. Relationships between alpha diversity and host factors were visualized with boxplots and scatterplots.

For beta-diversity analyses, microbiota data were converted to relative abundances to calculate Bray–Curtis distances or applied a centered log-ratio (CLR) transformation for Aitchison distances. A pseudocount of 0.5 was added prior to CLR transformation to account for zero values. Distance matrices were generated using the R *phyloseq* package. Associations between beta diversity and tortoise age (years), sex (male vs. female), normalized growth rate, and trophic category (herbivore vs. omnivore) were tested using PERMANOVA tests from the R *vegan* package (v.2.6–4) (Oksanen et al., 2022). A separate model evaluated the correlation between host species and beta-diversity. PCoA ordinations showed the clustering of samples color-coded by tortoise species, with shapes denoting their trophic category.

To further explore potential relationships between gut bacteria and tortoise growth rate, we conducted Spearman correlations. Specifically, we tested associations between growth rate (normalized by weight and age) and the relative abundances of 28 bacterial taxa. The selected taxa represented the most abundant taxa in the dataset (mean relative abundance >0.65% across samples) and included 15 taxa classified to species, 9 to genus, and 4 to family. *p*-values were adjusted for multiple comparisons using the False Discovery Rate (FDR). Significant associations were visualized using scatterplots of bacterial relative abundance versus tortoise growth rate.

To explore how the microbiotas of different tortoise species (red-footed, Russian, and leopard) varied, we performed differential abundance analyses using the R LinDA (v0.2.0) (Zhou et al., 2022) package. LinDA analyzed centered log-ratio (CLR)-transformed abundances. Two LinDA models were fit to capture all three pairwise species comparisons, with red-footed and Russian tortoises serving as the reference groups in model 1 and model 2, respectively. Analyses were restricted to bacterial taxa identified at the genus, or species level and present in at least 35% of samples from red-footed, Russian and leopard tortoises. Effect sizes for significant taxa were visualized using diverging plots generated in *ggplot2*.

## Results

Across seven captive *Testudine* tortoise species, a core gut microbiota consisting of 18 bacterial taxa was identified (Table 2). These bacterial taxa were detected in 70% of individuals, representing taxa present in more than two-thirds of individuals. The core included taxa previously associated with cellulose and fiber degradation (e.g., *Cellulosilyticum ruminicola*, *Ruminococcus champanellensis*, *Treponema* sp., and unclassified *Oscillospiraceae*), short-chain fatty acid production (e.g., *Clostridium butyricum*, unclassified *Lachnospiraceae*, and *Christensenellaceae*), protein degradation (*Clostridium* sp. and *Sarcina* sp.), and carbohydrate metabolism (*Clostridium saccharoperbutylacetonicum* and *Victivallis vadensis*; Table 2). These functional annotations provide context for the potential ecological roles represented within the core gut microbiota.

**Table 2:** Eighteen bacterial groups comprise the core microbiota across 7 species of captive tortoises.

Bacterial group	prev.	mean	median	min^*^	max	stdev	low10pct	pct50	high90pct	high97.5pct
Bacilli UC	1.000	2.712	1.452	0.133	13.134	3.238	0.304	1.452	8.305	11.734
Lachnospiraceae UC	1.000	2.009	1.213	0.154	14.206	2.254	0.424	1.213	3.579	7.528
Sarcina UC	1.000	14.148	10.232	0.385	56.374	12.764	3.627	10.232	29.623	51.695
Clostridium UC	0.982	7.632	4.916	0.134	32.585	7.590	0.456	4.916	18.984	25.264
Bacteroidales UC	0.964	11.184	7.689	0.106	49.397	11.683	0.307	7.689	23.993	42.211
Pirellulaceae UC	0.964	4.867	4.399	0.172	17.741	3.980	0.396	4.399	9.963	13.120
*Victivallis vadensis*	0.964	3.348	1.940	0.073	17.468	4.201	0.187	1.940	6.259	16.817
Christensenellaceae_R-7 UC	0.909	0.922	0.743	0.045	5.112	0.983	0.050	0.743	1.917	3.454
Oscillospiraceae UC	0.909	0.832	0.544	0.080	4.112	0.940	0.086	0.544	1.915	3.612
Bacteroides UC	0.855	1.768	0.493	0.032	14.780	3.308	0.000	0.493	6.172	11.642
Clostridia UC	0.836	0.556	0.343	0.045	2.938	0.613	0.000	0.343	1.256	2.080
Anaerosporobacter UC	0.782	2.289	1.623	0.124	9.259	2.494	0.000	1.623	6.880	7.549
Treponema UC	0.764	3.247	0.793	0.060	27.730	5.262	0.000	0.793	8.554	16.203
Bacteroidia UC	0.745	4.519	2.302	0.247	29.602	6.409	0.000	2.302	11.653	23.421
*Clostridium butyricum*	0.745	2.673	1.647	0.075	18.834	3.625	0.000	1.647	6.880	11.371
*Ruminococcus champanellensis*	0.745	1.028	0.607	0.080	6.530	1.350	0.000	0.607	2.493	4.722
*Cellulosilyticum ruminicola*	0.709	1.114	0.574	0.085	6.821	1.582	0.000	0.574	3.039	5.910
*Clostridium saccharoperbutylacetonicum*	0.709	2.527	0.541	0.124	21.040	4.826	0.000	0.541	7.855	17.831

Shown above are pertinent relative abundance (%) metrics. A 70% prevalence threshold and mean relative abundance >0.5% was used to define the core.

UC, unclassified; prev., prevalence; stdev, standard deviation; pct, percentile.

^*^The minimum value after excluding zeros.

**Figure 1:**
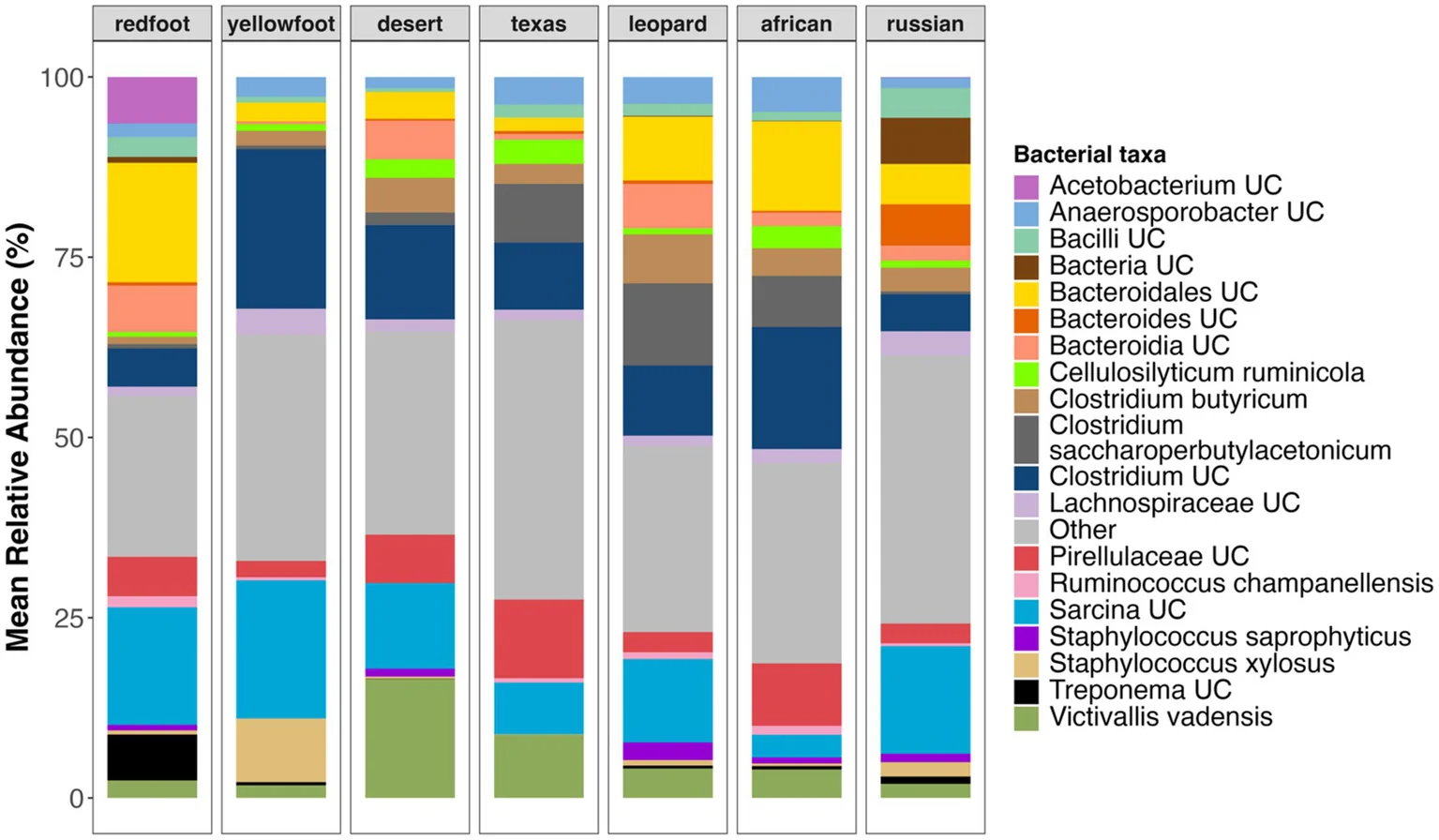
Gut microbiota composition across seven captive tortoise species. Stacked bars show the mean relative abundances of the most abundant bacterial taxa (mean relative abundance >1% across all samples) within each tortoise species. Remaining taxa are grouped into the “Other” category. UC (i.e., “Unclassified”) denotes taxa that could not be classified to the species level. Tortoise species appear in this order: red-footed (*N* = 25), yellow-footed (*N* = 2), Mojave Desert (*N* = 2), Texas (*N* = 2), leopard (*N* = 6), African-spurred (*N* = 4), and Russian (*N* = 14).

**Figure 2:**
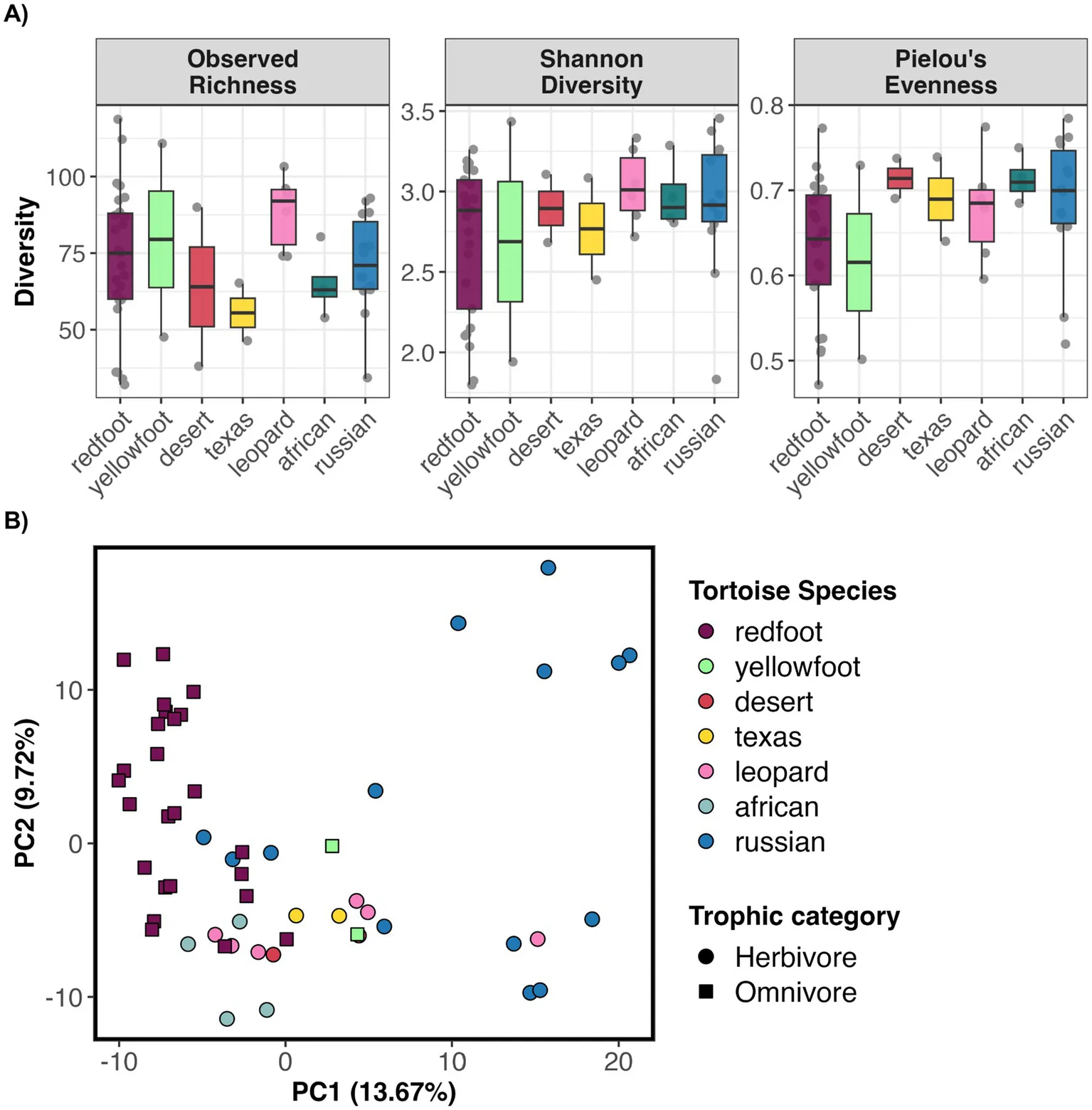
Host species is associated with gut microbiota beta-diversity but not alpha-diversity in captive tortoises. **(A)** Alpha diversity metrics (Observed Richness, Shannon Diversity, and Pielou’s Evenness) across seven tortoise species. Boxplots show the distribution of values among individuals within each species. **(B)** Principal coordinates analysis (PCoA) of microbiota beta diversity (Aitchison distance). Each point represents an individual tortoise, colored by host species and shaped by trophic category. Tortoise species appear in this order: red-footed (*N* = 25), yellow-footed (*N* = 2), Mojave Desert (*N* = 2), Texas (*N* = 2), leopard (*N* = 6), African-spurred (*N* = 4), and Russian (*N* = 14).

Although a common core microbiota was observed across tortoises, the relative abundance of these and other taxa varied across host species (Figure 1, Supplementary Table S3). Plots of gut microbiota composition show that African spurred tortoises for example, harbor more *Cellulosilyticum ruminocola* compared to the other species, leopard tortoises harbored more *C. butyricum*, and red-footed tortoises contained the largest abundances of *Acetobacterium* (Figure 1). Beta-diversity analyses confirmed this pattern and revealed that tortoise microbiotas cluster by host species (Figure 2B), with this factor accounting up to 25% of the variation in the microbiota (PERMANOVA Bray-Curtis *R*^2^ = 0.254, *p* = 0.001; Aitchison *R*^2^ = 0.232, *p* = 0.001). Restricting the analysis to the three tortoise species with the largest sample sizes (red-footed, leopard, and Russian tortoises) yielded similar results (PERMANOVA Bray-Curtis *R*^2^ = 0.168, *p* = 0.001; Aitchison *R*^2^ = 0.158, *p* = 0.001). Interestingly, microbiota richness and evenness did not differ by host species (Figure 2A; GLM LRT Observed Richness *F* = 0.96, *p* = 0.46; Shannon Diversity *F* = 0.81, *p* = 0.56; Pielou’s Evenness *F* = 1.5, *p* = 0.19). Thus, all captive tortoises regardless of species harbored microbiotas of comparable diversity.

**Figure 3:**
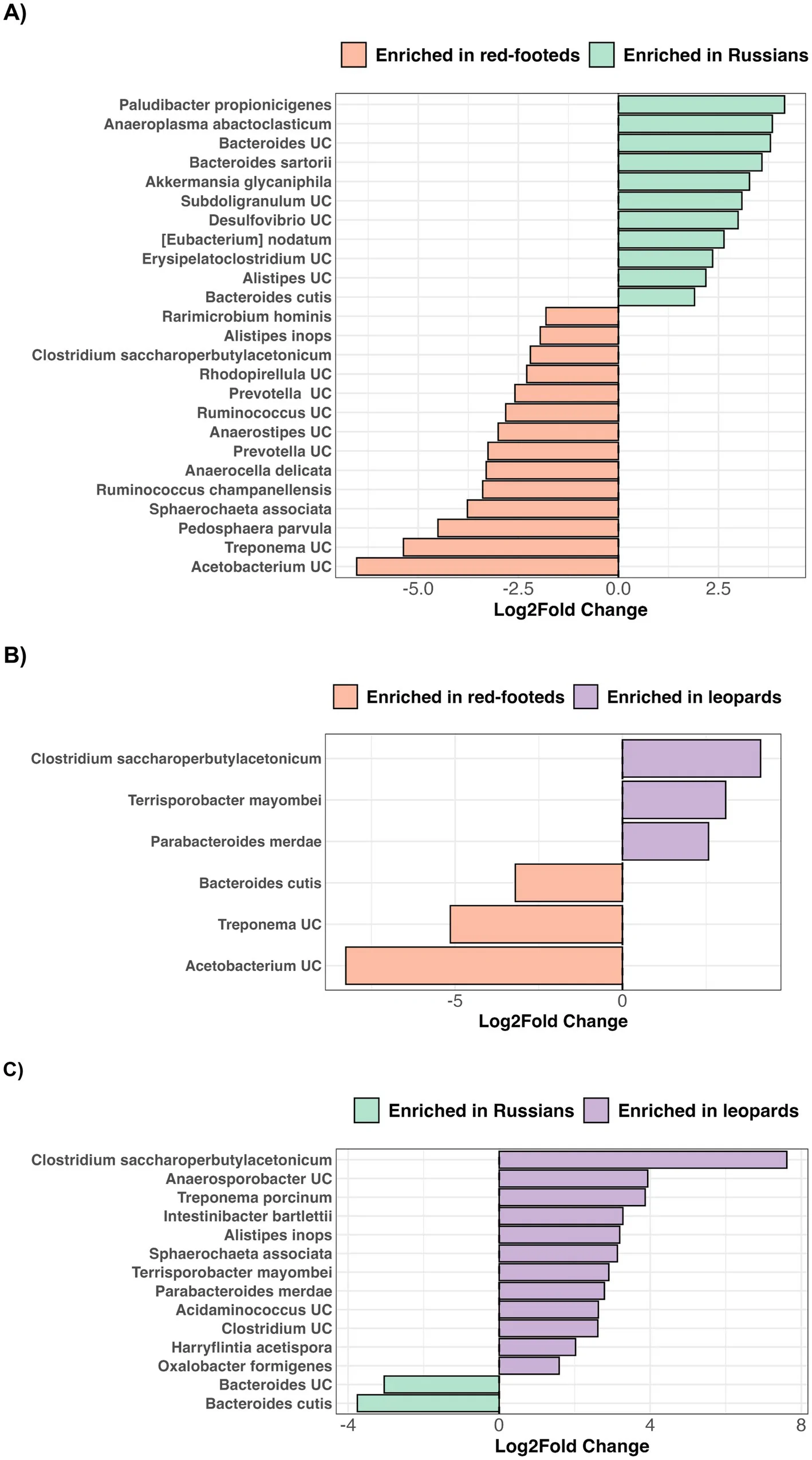
Bacterial species enriched in the gut microbiotas of red-footed (*N* = 25), leopard (*N* = 6), and Russian tortoises (*N* = 14). Results from LinDA differential abundance analyses performed at the bacterial species level. The LinDA models included host species as a categorical variable. **(A)** red-footed vs. Russian tortoises, **(B)** red-footed vs. leopard tortoises, and **(C)** Russian vs. leopard tortoises. UC denotes taxa that could not be classified to the species level.

To further resolve species-level differences in microbiota composition, we performed differential abundance analyses, focusing on the three tortoise species with the largest sample sizes. LinDA analyses indicated that the microbiota of red-footed tortoises were enriched in *Prevotella*, *Anaerostipes*, *Ruminococcus*, and *Treponema* compared to Russian tortoises, which instead contained more *Bacteroides*, *Desulfovibrio*, and *Paludibacter propionicigenes* (*p* < 0.05, Figure 3A, Supplementary Table S4). Furthermore, compared to red-footed tortoises, leopard tortoises were enriched in *C. saccharoperbutylacetonicum*, *Terrisporobacter mayombei*, and *Parabacteroides merdae* (Figure 3B, Supplementary Table S4). Russian tortoises contained more *Bacteroides cuti*s but less *Sphaerochaeta associata* (Figure 3C, Supplementary Table S4) than leopard tortoises.

**Figure 4:**
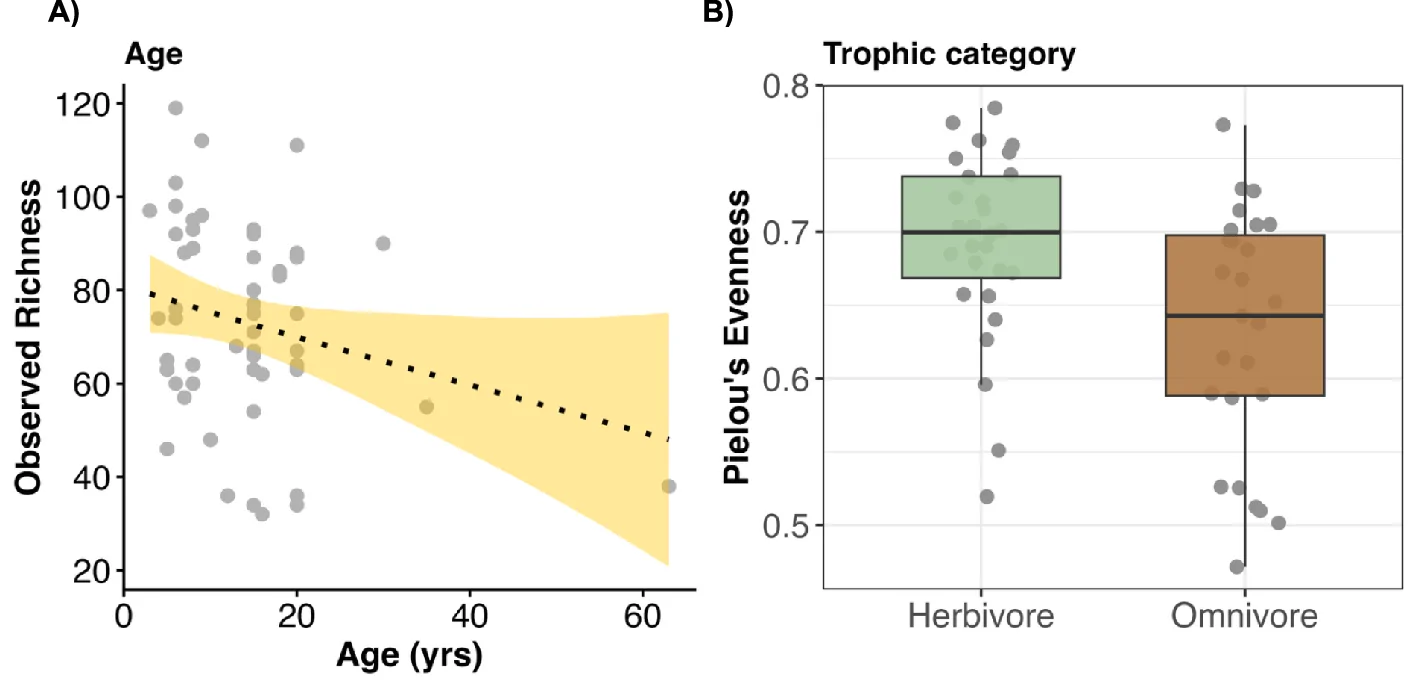
Gut microbiota alpha-diversity varies with tortoise age and trophic category. **(A)** Microbiota richness (observed richness) plotted against tortoise age (years). **(B)** Microbiota evenness (Pielou’s evenness) between herbivorous (*N* = 28) and omnivorous tortoises (*N* = 27).

Next, we examined whether other tortoise characteristics such as age, sex, growth rate (normalized by age and weight), and trophic category were associated with microbiota alpha- or beta-diversity. We found that microbiota richness was negatively associated with tortoise age (GLM *β* = −0.01 ± 0.005, *p* = 0.044), such that older tortoises had less diverse microbiotas than younger tortoises (Figure 4A). Microbiota evenness also differed by trophic category, with herbivores showing greater diversity (Shannon *x̄*: 2.96; Pielou’s Evenness *x̄*: 0.69) than omnivores (Shannon *x̄*: 2.69; Pielou’s Evenness *x̄*: 0.63; GLM LRT Shannon Diversity *F* = 5.39, *p* = 0.024; Pielou’s Evenness *F* = 8.28, *p* = 0.005; Figure 4B). Microbiota alpha-diversity was not significantly correlated with tortoise growth rate or sex (GLM *p* > 0.05). By contrast, analysis of microbiota beta-diversity indicated that microbiota profiles were significantly associated with tortoise sex, growth rate, and trophic category (PERMANOVA *p* < 0.05, Supplementary Table S5), though effect sizes were modest. Trophic category accounted for 8% of the variation in the microbiota, while sex and growth rate accounted for <3% of the variance each (Supplementary Table S5).

**Figure 5:**
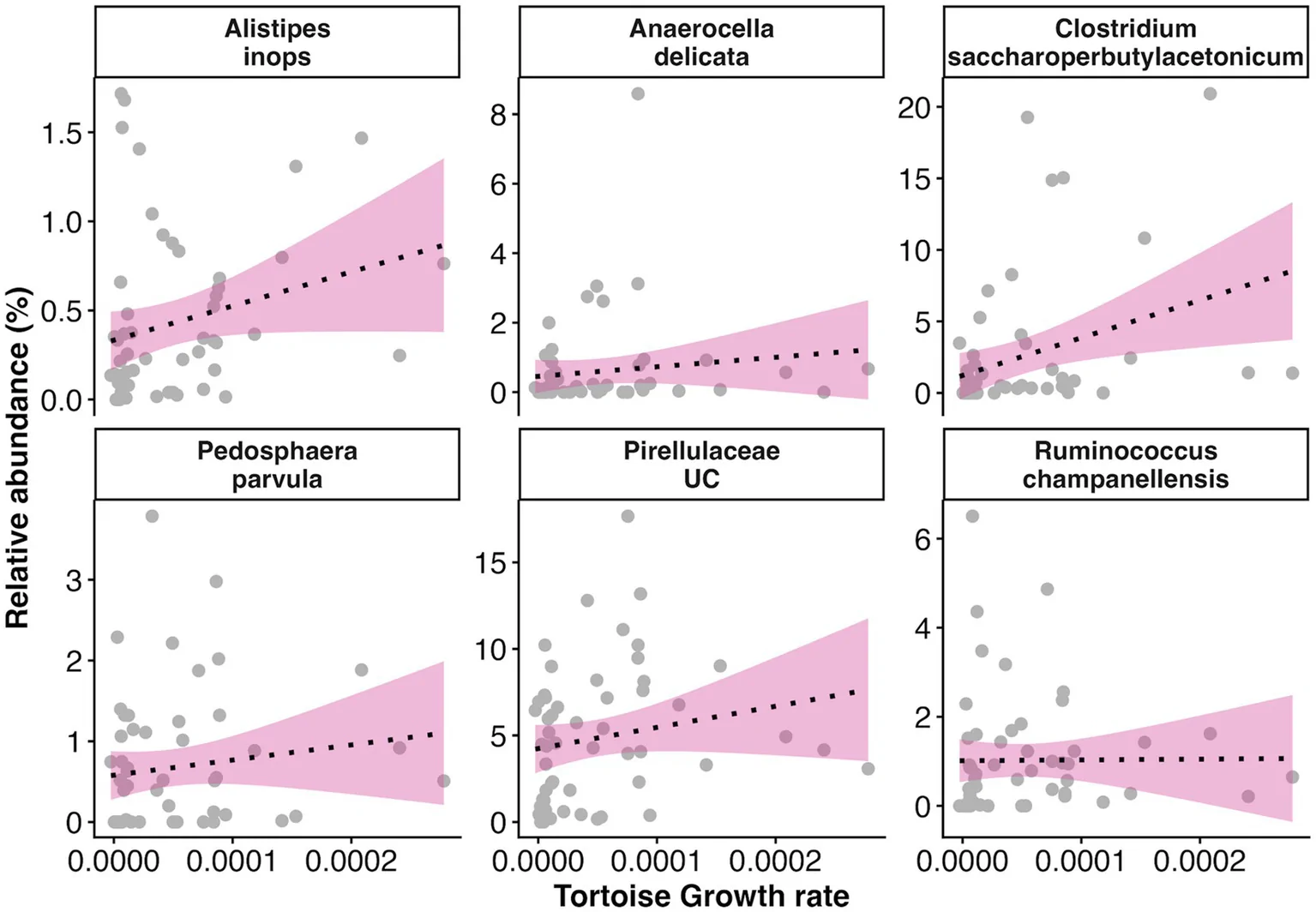
Bacterial taxa associated with tortoise growth rate. The six taxa shown below were significantly associated with tortoise growth by Spearman correlation analysis (FDR-adjusted *p* < 0.05). Solid lines represent fitted linear regression lines. UC denotes taxa that could not be classified to the species and genus level.

To further investigate the association between growth rate and microbiota beta-diversity, we examined differences in microbiota composition between faster- and slower-growing tortoises. For this, we correlated the relative abundances of 28 bacterial species with tortoise growth rate. We found that tortoise growth rate was positively associated with 6 of the 28 bacterial taxa examined, including *Anaerocella delicata*, *Alistipes inops*, *C. saccharoperbutylacetonicum*, *Pedosphaera parvula*, *Pirellulaceae* UC, and *R. champanellensis* (Spearman correlations *p* adjusted <0.05, Figure 5, Supplementary Table S6). Collectively, these results point to specific microbial taxa that may be associated with growth in tortoises. These associations do not imply functional causation and may reflect correlated ecological or host physiological processes.

## Discussion

This study examined gut bacterial microbiota variation in a multi-species population of captive tortoises maintained within a shared environment, allowing us to reduce large-scale environmental variability and more clearly evaluate association with host characteristics.

We found that tortoise species shared a common bacterial core microbiota composed of *Cellulosilyticum* sp., *Ruminococcus* sp., *Treponema* sp., unclassified *Lachnospiraceae*, and *Christenellaceae*. Many of these bacterial taxa have also been detected in other tortoise species including gopher tortoise (*Gopherus polyphemus*), Aldabra giant tortoise (*A. gigantea*), and Bolson tortoise (*Gopherus flavomarginatus*) (Zakaria et al., 2025; García-Dela Peña et al., 2019; Giakoumas et al., 2024), Beal’s eyed turtle (*Sacalia bealei*) (Fong et al., 2020), and even other hindgut fermenters like horses (Di Pietro et al., 2021) and zebras (Rojas et al., 2021). These bacterial taxa are associated with fiber degradation and fermentation (Blair et al., 2025; Paster and Canale-Parola, 1985; Cwyk and Canale-Parola, 1979; Cai et al., 2010; Chassard et al., 2012), highlighting the potential importance of microbial metabolism in tortoise nutrition. Tortoises are hindgut fermenters whose diet comprises fermentable, plant-derived starches, such as cellulose and hemicellulose (Bjorndal, 1989; Hatt et al., 2005). These starches are metabolized by gut bacteria and the metabolites are converted into energy and heat by their host. In-vitro studies of microbial communities isolated from desert tortoise (*Gopherus agassizii*) fecal samples show that taxa such as *Lachnospiraceae* thrive in lignocellulose-rich enrichments and produce short-chain fatty acids under anaerobic conditions (Blair et al., 2025). Although the shared captive environment likely contributes to a common core microbiota, the widespread occurrence of these taxa across herbivorous vertebrates suggests that host digestive physiology may also contribute to their persistence (Ley et al., 2008; Muegge et al., 2011).

At the same time, this bacterial core was not uniform, and variation in the presence and relative abundance of taxa was observed across individuals. Host-associated factors, particularly species and trophic category, were strongly associated with differences in microbial composition, whereas age had comparatively limited effects. This pattern aligns with a substantial body of literature demonstrating that host species is a primary determinant of gut microbiota structure across vertebrates (Ley et al., 2008; Amato et al., 2019; Youngblut et al., 2019). Similar species-level structuring has been observed in reptiles, where host phylogeny and associated physiological differences contribute to distinct microbial communities even under shared or overlapping environmental conditions (Colston and Jackson, 2016; Kohl et al., 2017). It is interesting to note that even in captivity, species-specific microbiota signatures emerge, echoing prior findings (McKenzie et al., 2017). At the same time, diet, feeding patterns, life history, and other adaptive traits are intertwined with species, limiting the ability to fully disentangle their independent effects on microbiota composition. Furthermore, although animals were maintained under broadly consistent husbandry practices, species-specific enclosures and microenvironmental differences may have contributed to variation in microbiota composition.

In our study, host factors collectively accounted for ~39% of the variation in the microbiota, leaving much of the variance unexplained. Such variability is likely driven by additional processes influencing microbiota assembly, including early-life microbial exposure (Carranco et al., 2022), competition during colonization (Costello et al., 2012), and subtle differences in host behavior or microenvironment (Zakaria et al., 2025; Harlow et al., 2025). This pattern is likely influenced by the sanctuary setting (McKenzie et al., 2017), where individuals often have diverse and unknown life histories that may shape initial microbiota assembly. Early colonization events can have lasting effects on community structure, where initial microbial populations influence subsequent community development and resist later colonization (Fukami, 2015). In addition, host-associated factors such as gut physiology and immune interactions (Robertson et al., 2018) can promote stability of established communities, contributing to persistent differences among individuals over time. Together, these results suggest that species and diet-related variables may contribute to broad compositional structure, while individual-specific factors may underlie finer-scale microbiota differences.

Importantly, variation in microbiota composition was associated with differences in growth among individuals in this study. Because this study is observational, we cannot determine the directionality of the observed association. Differences in microbiota composition could contribute to variation in growth, reflect underlying host physiology, or both. However, in hindgut-fermenting herbivorous species—and in omnivorous species where plant material constitutes a substantial portion of the diet—gut microbes are known to contribute to the fermentation of fibrous plant material into short-chain fatty acids, which can serve as a major energy source for the host (Bjorndal, 1989; Mackie, 2002; Morrison et al., 2009). Differences in microbiota composition could influence fermentation efficiency and energy extraction, providing one plausible mechanistic explanation between microbial community structure and growth. Similar associations have been observed in reptiles, including Reeves’ turtles (*Mauremys reevesii*), where differences in gut microbiota composition and specific microbial taxa have were associated with variation in growth performance and digestive capacity (Xu et al., 2025). Associations between the gut microbiota and growth have also been reported in Svalbard reindeer (Kamenova et al., 2025), although in that system the relationship is strongly influenced by environmental changes such as Arctic greening.

It is important to note that the bacterial taxa positively associated with tortoise growth rate were not all cellulose degraders. *R. champanellensis* is a well-known fiber-degrading specialist (Chassard et al., 2012), whereas *A. delicata* and *C. saccharoperbutylacetonicum* are involved in the breakdown of dietary sugars and carbohydrates, fermenting them into short-chain fatty acids such as butyrate and acetate (Abe et al., 2012; Baur et al., 2022). Members of the family *Pirellulaceae* (phylum Planctomycetes) have been primarily isolated from sediments, lakes, and seawater (Vitorino et al., 2022; Kulichevskaya et al., 2022; Sreya et al., 2023), where they are involved in carbon cycling, and in tortoises, they may contribute to the breakdown of recalcitrant plant material.

From a practical perspective, these findings may help explain a familiar observation in captive tortoise husbandry. While environmental and husbandry factors clearly influence tortoise growth, and captive tortoises exhibit greater morphological variability than their wild counterparts (Ritz et al., 2010; Semaha et al., 2025), substantial differences in growth among individuals maintained under broadly similar captive conditions is also a common observation among rescue organizations, breeders, and hobby keepers. Our findings suggest that differences in gut microbial communities may represent one previously unrecognized contributor to this residual variation. This does not imply that the microbiota is the primary determinant of growth, but rather that it may contribute to the unexplained variability observed among individuals.

## Limitations

Our study has several limitations that should be considered when interpreting our findings. First, because this was an observational study, we were not able to determine the direction of the association between microbiota composition and growth rate. Experimental studies that manipulate the gut microbiota will be needed to determine whether microbial communities contribute to host growth. Studies that measure growth rate over many years and sample the microbiota at multiple time points will also be key. Second, we measured growth rate as body weight normalized by age rather than repeated measurements of carapace length. Future studies employing direct measurements of tortoise growth by measuring carapace length, plastron length, and tortoise height would provide greater insight into microbiota–growth relationships. Third, trophic category was classified broadly as herbivorous or omnivorous, and more detailed dietary measurements, such as stable isotope analyses or quantified diet records, would provide a more refined assessment of diet-microbiota associations. Finally, sample sizes were unbalanced across species, with several species represented by only two individuals. Broader sampling across additional tortoise species will improve statistical power and help determine the generality of these findings.

## Conclusion

This study demonstrates that tortoises maintained within a shared captive environment harbor a conserved core gut microbiota associated with fiber degradation and fermentation, while still exhibiting substantial variation in community composition among individuals. Differences in microbiota composition were associated with host species and diet, although these factors did not fully explain the observed variability. Variation in growth rate was also associated with differences in microbial composition, highlighting the potential relationship between the gut microbiota and host growth.

Together, these findings highlight the gut microbiota as an additional and previously underappreciated source of biological variation in captive tortoises. In practical terms, this may help explain why individuals maintained under similar husbandry conditions can exhibit markedly different growth outcomes, emphasizing the importance of considering microbiota variation alongside traditional factors when interpreting and managing host health and performance. Future longitudinal or experimental studies will be needed to determine whether and how targeted manipulation of the gut microbiota can influence growth outcomes in tortoises.

Our findings help address key gaps in the literature, as the gut microbiota of tortoises particularly across multiple species remains poorly characterized, and research in these reptiles is still in its early stages.

## Data Availability

Raw PacBio Hifi sequences (.fastq) are available on the NCBI SRA under BioProject PRJNA1454561, and SRA accessions SRR38193976 - SRR38194030.
